# Gilles de la Tourette Syndrome and Habit-Reversal: A Case Report

**DOI:** 10.1192/j.eurpsy.2025.2152

**Published:** 2025-08-26

**Authors:** D. Patel, H. Tyagi

**Affiliations:** 1 Department of Neuropsychiatry, University College London Hospital NHS Foundation Trust, London, United Kingdom

## Abstract

**Introduction:**

Gilles de la Tourette Syndrome (GTS) is characterised by tics which appear as sudden, rapid, purposeless motor movements and vocalisations. In contrast to other movement disorders, temporary and purposeful suppressibility for a few minutes at a time can be achievable. However, this is ineffective over time. Apart from the physical consequences incurred, tics and their associated neuropsychiatric symptoms can diminish individual quality of life.

**Objectives:**

To present an adult single case study of the implementation of Habit Reversal Training (HbRT) for the treatment of a motor tic and to determine the clinical efficacy of the intervention over time (i.e., post-intervention and at a one-, three- and six-month follow-up).

**Methods:**

A twenty-six-year-old male patient with a well-established diagnosis of GTS was referred to a tertiary-care neuropsychiatry outpatient clinic. Prior to the HbRT intervention, the patient had well-tolerated a continuous tetrabenazine prescription (25mg twice a day). His tic consisted of twitching of his nostrils and sudden and repeated head nods. The tic was reported to being experienced throughout the day and almost always being preceeded by a premonitory sensation. The patient’s history was unremarkable with respect to pre, peri-, and postnatal development. There was no family history of tics nor any other movement disorders. Formal measures revealed the following: Clinical Outcomes in Routine Evaluation-Outcome Measure (**11**), Frost Multidimensional Perfectionism Scale (**117**), Autism-Spectrum Quotient (**2**), Adult Attention Deficit Hyperactivity Disorder Scale (**1**), and Yale-Brown Obsessive Compulsive Scale (**15).**

**Results:**

By the end of a five-week fifty-minute one-to-one intervention window and at a one-, three-, and six-month follow-up appointment, the following main results are reported: [i] at post-intervention, a self-reported tic improvement score (measuring effectiveness of competing response on tic management since the first appointment) of eighty percent was achieved, [ii] self-reported tic improvement scores carried over to all three follow-up appointments, and [iii] week-to-week monitoring revealed that tic management improved by fifty percent by the third week of the intervention.

**Conclusions:**

This study has accomplished its objectives of offering additional support for the implementation of HbRT for the treatment of a motor tic and to establish the clinical efficacy of the intervention over time. With these objectives in mind, TD and GTS continues to provide clinicians, clinician-scientists, and researchers with an abundance of possibilities for future research. For instance, on a clinical level, it is essential to further characterize variations in motor tic phenotype so that the factors that modify tic behaviour can be clarified. It would also be fascinating to longitudinally study and explore changes in tic frequency and intensity over time following a behavioural intervention such as HbRT.

**Disclosure of Interest:**

None Declared

